# The Backstroke Swimming Start: State of the Art

**DOI:** 10.2478/hukin-2014-0058

**Published:** 2014-10-10

**Authors:** Karla de Jesus, Kelly de Jesus, Ricardo J. Fernandes, João Paulo Vilas-Boas, Ross Sanders

**Affiliations:** 1 Centre of Research, Education, Innovation and Intervention in Sport, Faculty of Sport, University of Porto, Porto, Portugal.; 2 Porto Biomechanics Laboratory, University of Porto, Porto, Portugal.; 3 Centre for Aquatics Research and Education, Institute for Sport, Physical Education, and Health Sciences, The University of Edinburgh, Edinburgh, UK.; 4 Exercise and Sport Science, Faculty of Health Sciences, The University of Sydney, Sydney, Australia.

**Keywords:** Biomechanics, dorsal starts, starting technique, starting variant, literature review

## Abstract

As sprint swimming events can be decided by margins as small as .01 s, thus, an effective start is essential. This study reviews and discusses the ‘state of the art’ literature regarding backstroke start biomechanics from 23 documents. These included two swimming specific publications, eight peer-reviewed journal articles, three from the Biomechanics and Medicine in Swimming Congress series, eight from the International Society of Biomechanics in Sports Conference Proceedings, one from a Biomechanics Congress and one academic (PhD) thesis. The studies had diverse aims, including swimmers’ proficiency levels and data collection settings. There was no single consensus for defining phase descriptions; and kinematics, kinetics and EMG approaches were implemented in laboratory settings. However, researchers face great challenges in improving methods of quantifying valid, reliable and accurate data between laboratory and competition conditions. For example, starting time was defined from the starting signal to distances as disparate as ∼5 m to 22.86 m in several studies. Due to recent rule changes, some of the research outcomes now refer to obsolete backstroke start techniques, and only a few studies considered the actual international rules. This literature review indicated that further research is required, in both laboratory and competition settings focusing on the combined influences of the current rules and block configuration on backstroke starting performances.

## Introduction

The total swimming race time is the sum of the starting, stroking and turning times (Guimarães and Hay, 1985). The start is the swimming race fastest part (Thow et al., 2012) and, if performed effectively, can influence race finishing position (Arellano et al., 2003; Cossor and Mason, 2001; Girold et al., 2001; Thanopoulos et al., 2012). In fact, nearly all the small temporal differences in the short distance events (i.e., 50 m and 100 m) might be explained by the starting efficiency (Ikuta et al., 2001). For instance, at 15 m after the start, the second-place finisher of men’s 100 m backstroke at Barcelona 2013 Swimming World Championships was 0.20 s slower than the eventual winner, and the final race time difference was 0.19 s. The importance of the start is emphasized further in that the time differences between individual international level swimmers at 15 m after the start can vary by 0.30 s in the same race (Vantorre et al., 2010).

Backstroke is the only competitive swimming technique in which the swimmer starts in the water. In accordance with the backstroke start rules at the Federation Internationale de Natation (FINA) from earlier 1960s to 2005, swimmers grasped the handgrips and placed their entirely immersed feet on the wall. Gripping one’s toes on the pool gutter was not allowed. FINA backstroke start rules for feet positioning were modified by the National Collegiate Athletic Association (NCAA) from the early 1960s to 1990 to allow swimmers to curl their toes over the starting wall gutter. However, from 1991 to 2006 the feet positioning was restricted to underwater. This modification was made to prevent injuries in competitive swimming involving backstroke starts (Cornett et al., 2011). From 2005, FINA established that swimmers must position their hands on the starting grips and their feet totally or partially immersed or entirely out of the water without using the gutter (SW 6.1, FINA, 2005–2009). The alleged advantages of feet placed high on the wall to generate greater horizontal take-off velocity (de Jesus et al., 2011a; 2013; Nguyen et al., 2014), vertical peak force (Nguyen et al., 2014), and consequently faster start times (Nguyen et al., 2014), might be considered the main reason for the respective rule adaptation. After the 2008 Olympic Games, the FINA approved a new designed starting block (OSB11, Corgémont, Switzerland), which included a back plate and three different backstroke start handgrips (i.e., two horizontal and one vertical) (FR 2.7, FINA 2009–2012). Recently, a non-slip wedge was authorised by FINA for feet placement during backstroke starts (FR 2.7, FINA, 2013–2017).

Despite the controversies between ruling authorities, and considerable swimming and facility backstroke start rule changes recently authorized by FINA, researchers have mainly attempted to analyse the ventral start biomechanics (e.g. Takeda et al., 2012). The greater quantity of ventral start studies is firstly justified by the greater quantity of events that begin from a starting block rather than in water (Theut and Jensen, 2006). Also, prior to recent rule changes, some controversies were possible with the dorsal, in-water start positions performed under the FINA rules (Vilas-Boas and Fernandes, 2003) and the difficulties concerning the underwater experimental set-up arrangements. Cornett et al. (2011) mentioned the non-existence of documented catastrophic injuries in competitive swimming backstroke starts as one reason for the scarce research. The backstroke start has been considered a more difficult and complex movement than the ventral techniques (de Jesus et al., 2011a; 2013; Nguyen et al., 2014; Takeda et al., 2014). It involves different skills to achieve the mechanical goals (de Jesus et al., 2011a; 2013; Maglischo, 2003; Nguyen et al., 2014; Takeda et al., 2014) and more scientific evidence is required.

The importance of swimming starts for enabling backstrokers to improve overall performances due to swimming rule changes and starting block modifications, makes it a valuable process to synthesise the scientific knowledge relating to backstroke starts. Literature reviews published regarding ventral start techniques were conducted by Vilas-Boas and Fernandes (2003) and Vantorre et al. (2014). This paper reviews the ‘state of the art’ regarding the biomechanics of backstroke starts. It underscores the gaps in and limitations of existing knowledge, and presents topics for future research to enable coaches and swimmers to better refine backstroke start training.

## Material and Methods

### Search strategy

The literature search was performed using PubMed, SportDiscus™, Scopus and ISI Web of Knowledge electronic databases, only for English written documents published before March 2014. Key words including “swimming”, “backstroke” and “start” were used to locate documents. Besides the electronic databases, the identified reference lists in the articles were also used to ensure, as far as practically possible, that all appropriate studies were considered for inclusion. Searches were carried out from the Proceedings of the Scientific Conferences of Biomechanics and Medicine in Swimming (BMS), the International Society of Biomechanics in Sports (ISBS), and the International Society of Biomechanics (ISB) from 1980 to 2013.

### Inclusion and exclusion criteria

Included studies were experimental biomechanical approaches in the laboratory or during competitions with able-bodied swimmers. The documents that were available only as abstracts and duplicated studies from original investigations were excluded.

## Results and Discussion

### General Findings

Eighty-seven references were obtained from the preliminary search. Ultimately, 23 studies met the inclusion criteria: (i) two from swimming specific journals; (ii) eight peer-review journal articles; (iii) three from the proceedings of the BMS conferences; (iv) eight from proceedings of the ISBS conferences; (v) one from proceedings of an ISB Biomechanics Conference, and (vi) one doctoral thesis (Table 1).

Table 1 reveals a large variation in experimental designs that were used. Most of the studies analysed the different backstroke start variations performed under FINA rules (86.5%). Overall, studies included Olympic, International and National backstroke swimmers, who were able to master the aspects of the already tested backstroke starting techniques. The research settings included laboratory and competition analyses performed in the Commonwealth Games (Miller et al., 1984), Olympic Games (Arellano et al., 2001; Cossor and Mason, 2001; Chatard et al., 2003; Girold et al., 2001; Ikuta et al., 2001), Youth Olympics (Arellano et al., 2003), Age Group Swim Meeting (Cornett et al., 2011), and European Championships (Siljeg et al., 2011). The biomechanical settings in high calibre events might be more advantageous than the laboratorial conditions to obtain valid performance outcomes (Toubekis et al., 2013; Schwameder, 2008). Otherwise, the competition rules often hamper the use of biomechanical methodology, thereby narrowing the possibility of obtaining accurate and reliable data (Schwameder, 2008).

The above mentioned factors, along with a limited number of existing studies, restrict quantitative assessments of the backstroke start variables. Therefore, a qualitative description was developed on relevant backstroke start evidence. This included the separate features of the starting phases, the biomechanical approaches used, and the start techniques and variations for which the main findings have been reported.

### Backstroke starting phases

#### Aerial

**Figure 1:**
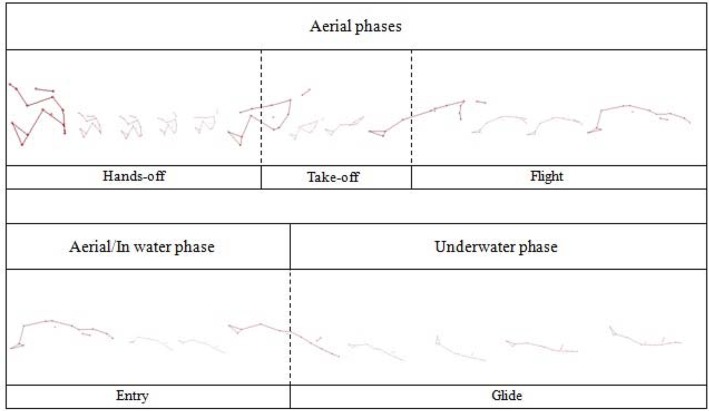
The most common starting phases and respective initial and final instants reported in the included studies, the starting signal, swimmer’s hands-off, swimmer’s feet take-off, swimmer’s fingertip water contact, swimmer’s full body immersion and beginning of lower limbs propulsive movements

The hands-off, take-off and flight are the most common aerial starting phases studied (Figure 1). However, the respective descriptions vary in the literature, with disparities that hamper communication among biomechanists, coaches and swimmers. In fact, breaking down a swim-start into its component parts can be challenging as each phase is not always clear cut (Vantorre et al., 2014). The hands-off and take-off phases are characterised by actions performed when swimmers are in contact with the starting wall. The beginning of the hands-off phase is determined by the starting signal (Figure 1) (de Jesus et al., 2011a; 2013; Green, 1987; Hohmann et al., 2008; Miller et al., 1984) and the swimmer’s first observable movement (Hohmann et al., 2008). Considering the take-off phase, authors determined the starting signal (Cossor and Mason, 2001; Hohmann et al., 2008; Miller et al., 1984; Nguyen et al., 2014; Stratten, 1970; Takeda et al., 2014), and the hands-off (de Jesus et al., 2010; 2011a; 2011b; 2013; Green, 1987; Hohmann et al., 2008) (Figure 1) as the instant of the beginning phase. This was also observed in ventral start studies (Takeda et al., 2012; Thanopoulos et al., 2012; Vantorre et al., 2010), where the hands-off was less analysed than the take-off in backstroke start studies.

The beginning of the flight phase was unanimously described as the instant of take-off by the feet (Cossor and Mason, 2001; de Jesus et al., 2011a; 2013; Green, 1987; Hohmann et al., 2008; Miller et al., 1984; Nguyen et al., 2014; Takeda et al., 2014) (Figure 1). However, authors differed regarding the conclusions for flight. These included: the instant that the head contacted the water (Cossor and Mason, 2001; Nguyen et al., 2014), the instant of the hip entry (Hohmann et al., 2008) and fingertip water contact (de Jesus et al., 2010; 2011a; 2013; Green, 1987; Miller et al., 1984; Takeda et al., 2014) (Figure 1). According to Maglischo (2003), the fingertip water contact is widely used to determine the end of the flight phase (Vantorre et al., 2014). The head and/or fingertip water contact could be a more appropriate reference point than the hip entry, since swimmers could immerse the hips before the hands/head contact the water (Takeda et al., 2014).

#### Aerial/In water and underwater phases

The entry and glide are the commonly studied aerial/in-water and underwater phases, respectively (Figure 1). As previously reported in ventral start studies, these phases have been less analysed than the aerial phases, even though they contribute to reaching a considerable distance from the wall at the beginning of a race (Vantorre et al., 2014). Further, contradictory definitions were found for some specific points of measurement.

The beginning of the entry phase corresponds to the final instant of the flight; and, for which, definitions differ among authors (de Jesus et al., 2011a; Green, 1987; Hohmann et al., 2008). The end of the entry phase is defined as the maximum feet depth from the first downward underwater kicking by Hohmann et al. (2008) but the full body immersion by de Jesus et al. (2011a) and Green (1987). Full body immersion is considered to be the end of the entry phase in ventral start studies (Vantorre et al., 2010) (Figure 1).

Authors have defined the glide phase as beginning at the instant entry ends until the maximum feet depth of the second downward underwater kick is reached (Hohmann et al., 2008), the hands reach the 5 m mark (de Jesus et al., 2011a; 2013), and/or the instant before underwater kicking commences (Green, 1987). In competition, Miller et al. (1984) defined the glide phase as being from when the fingertips made first water contact, until the first hand which came out of the water at the end of the glide, re-enters the water. Cossor and Mason (2001) considered the entry, glide and undulatory underwater movements as one combined parameter.

In previous ventral start studies, authors divided the underwater phase into two parts: the glide (Guimarães and Hay, 1985; Thow et al., 2012; Vantorre et al., 2010) and the undulatory underwater swimming (Vantorre et al., 2010). This convention was adopted by de Jesus et al. (2012) for the backstroke start. The glide phase does not include lower limb propulsive movements (Guimarães and Hay, 1985; Thow et al., 2012; Vantorre et al., 2014) (Figure 1). Hence, future studies should examine if the underwater kicking observed by Hohmann et al. (2008) as soon as the feet entered the water, provides any advantage over a period of motionless gliding during the start.

### Biomechanical approaches and parameters assessed

#### Kinematics

Despite some authors using immediate feedback devices such as stopwatches (Green et al., 1987; Stratten, 1970) and velocimeters (de Jesus et al., 2012), 82.6% of the studies assessed backstroke start kinematics using video-based techniques (Arellano et al., 2001; Arellano et al., 2003; Chatard et al., 2003; Cornett et al., 2011; Cossor and Mason, 2001; de Jesus et al., 2010; 2011a; 2013; Girold et al., 2001; Green, 1987; Hohmann et al., 2008; Ikuta et al., 2001; Miller et al., 1984; Nguyen et al., 2014; Rea and Soth, 1967; Siljeg et al., 2011; Takeda et al., 2014; Theut and Jensen, 2006; Wilson and Howard, 1983). Only Green (1987) used a three-dimensional (3D) dual-media setting via cinematographic cameras.

Most studies used digital cameras to provide independent aerial, underwater or combined dual-media analysis. In competition settings, cameras were positioned 18 m above the swimming pool (Arellano et al., 2001; Cossor and Mason, 2001; Girold et al., 2001; Ikuta et al., 2001) and along the side of the pool, 15 m from the starting block wall (Arellano et al., 2003); or underwater at 6.5 m from the starting block wall (Cornett et al., 2011). Studies conducted under laboratory conditions, used aerial and underwater cameras positioned at 6.78 m (de Jesus et al., 2010; 2011a; 2013) and 7.5 m (Takeda et al., 2014), both from the primary swimmer’s plane of motion, and 30 cm above- and below-water surface (de Jesus et al., 2010; 2011a; 2013). Takeda et al. (2014) also described the dual-media cameras as positioned above the pool side deck and 1 m below the water surface; while Theut and Jensen (2006) implemented the same above-water camera position but the underwater camera in the corner of the swimming pool. Hohmann et al. (2008) and Nguyen et al. (2014) did not provide further details about the dual-media camera positions.

Quantitative data processing from digital cameras usually requires a coordinate scale and prevents immediate results due to the need for manual digitising (de Jesus et al., 2011a; 2013; Hohmann et al., 2008; Nguyen et al., 2014; Takeda et al., 2014; Theut and Jensen, 2006). Furthermore, the digitisation and reconstruction errors associated with this procedure require authors to measure the errors. However, only de Jesus et al. (2011a; 2013) and Takeda et al. (2014) displayed these values. In competition settings, challenges increase because the competition regulations make it difficult to use the most accurate biomechanical methodology (Schwameder, 2008) which requires researchers to use parts of the swimming pool to create a digitising scale (Miller et al., 1984). The automatic tracking motion analysis systems have been considered highly reliable for 3D underwater analysis (Kudo and Lee, 2010). However, further validation and reliability testing is required to establish its viability for studying dual-media backstroke starts.

Most of the kinematics approaches mentioned in the backstroke start studies above provide biomechanical performance indicators instead of specifying how swimmers should organize body segments movements to optimise their performance. Performance indicators are less time-consuming for coaching feedback and hinder technique analysis method to be wide-used in backstroke start studies. Table 2 outlines the kinematic variables measured at the most common backstroke starting phases and for the overall start. In fact, 69.5% of the studies measured the starting time, which ranged from the signal to the first fingertip contact with the water (de Jesus et al., 2011a; 2013) and the time to 22.86 m (Green et al., 1987). Following Guimarães and Hay (1985), starting time has been often measured for ventral start studies (Vantorre et al., 2010), but, there is no clear consensus as to what distances are best for assessing the most effective start, yet.

Table 2 indicates that most backstroke start studies have measured only linear displacement and velocity parameters, despite swimming starts not being exclusively rectilinear motions (Bartlett, 2007). Authors have considered the swimmer as a rigid body to calculate the horizontal distance (Cornett et al., 2011; Cossor and Mason, 2001; Miller et al., 1984; Theut and Jensen, 2006) and the velocity during a backstroke start (Arellano et al., 2003; Chatard et al., 2003; Giroldi et al., 2001; Theut and Jensen, 2006). Although these variables provide important information in training and competition environments, the curvilinear motions in the backstroke start need to be quantified. Some authors have studied translational kinematic parameters of the centre of mass or hip vectors during the overall backstroke start (Green, 1987) and during starting phases (de Jesus et al., 2010; 2011a; 2013; Green, 1987; Nguyen et al., 2014; Takeda et al., 2014), as have been conducted for ventral starts (Guimarães and Hay, 1985; Takeda et al., 2012).

As humans do not have rigid bodies and display combinations of rotational and linear motions (Bartlett, 2007), multi-segmental models have been used to analyse segmental positions (Nguyen et al., 2014; Takeda et al., 2014); and joint angles from upper (Green et al., 1987; Wilson and Howard, 1983) and lower limbs (de Jesus et al., 2010; de Jesus et al., 2011a; Green et al., 1987; Nguyen et al., 2014; Takeda et al., 2014; Wilson and Howard, 1983); and trunks (de Jesus et al., 2013; Wilson and Howard, 1983) at different starting phases (Table 2). The study of the coupling relationship between segments is required to provide insight into the optimal movement strategies underlying backstroke starts.

There is a paucity of evidence concerning the parameters in the aerial/in-water and underwater phases. In fact, research usually has highlighted the importance of assessing entry (Vantorre et al., 2010; Vantorre et al., 2014) and underwater phase kinematics (de Jesus et al., 2011a; Vantorre et al., 2010; Vantorre et al., 2014; Thow et al., 2012) for ventral starts. Only Green (1987) and de Jesus et al. (2011a) have calculated the centre of mass displacement and velocity, during the entry and glide phases; and the time and frequency of some undulatory underwater swimming cycles of the backstroke start (de Jesus et al., 2012). In competitions, authors have measured the combined time from the entry until the swimmer’s head resurfaced (Cossor and Mason, 2001) and the beginning of the first arm stroking cycle (Miller et al., 1984).

#### Kinetics

Despite several studies having used kinematics, few studies of backstroke starts have included kinetic data. Kinetics requires higher costs than image based systems and presents technical difficulties when attaching the kinetic devices to the starting block and pool wall. However, de Jesus et al. (2010; 2011a; 2013) successfully lowered, then elevated pool water levels so as to position a strain gauge force plate at two heights on the pool wall. Also, they instrumented the handgrips with a strain gauge load cell which was sequentially repositioned to remain at the same distance above the water surface. The dynamics between the lower limbs and the pool wall were studied using a 3D piezoelectric force plate (Hohmann et al., 2008; Nguyen et al., 2014). The strain gauges are more commonly used due to their lower costs and highly accurate static and transient load measurement capabilities than via a 3D piezoelectric force plate.

**Figure 2:**
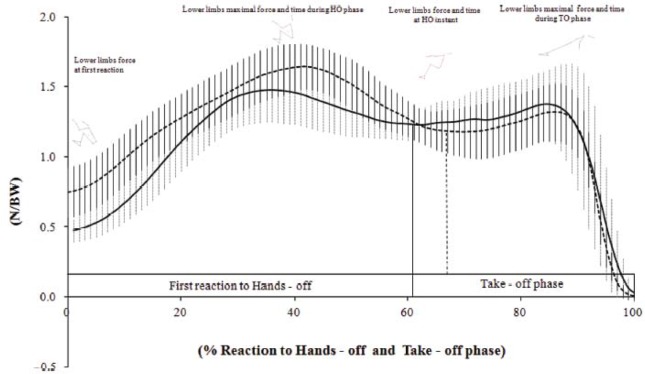
Mean lower limbs horizontal force-time curves for backstroke start with feet immerged (continuous line) and emerged (dashed line) (de Jesus et al., 2013)

The instrumentation of starting blocks for analysing backstroke starts has helped to verify how the respective movements are generated (de Jesus et al., 2013; Hohmann et al., 2008; Nguyen et al., 2014). The horizontal force exerted by swimmers’ lower limbs on the pool wall is the main research topic of backstroke start kinetics (de Jesus et al., 2013; Hohmann et al., 2008; Nguyen et al., 2014). The horizontal swimmers’ lower limbs force-time curve profiles (Figure 2) registered during backstroke start performances were similar among these three studies reporting two distinguished peak forces. Researchers stated that swimmers should optimise the force-time distribution during the take-off phase (de Jesus et al., 2011a; 2013; Guimarães and Hay, 1985; Hohmann et al., 2008; Nguyen et al., 2014; Vantorre et al., 2014). To obtain further insight into the mechanics of the backstroke start, de Jesus et al. (2011a; 2013) analysed the horizontal forces exerted on the handgrips and noted that the role played by the upper limbs was to drive the centre of mass above the water surface.

Despite the understanding about the horizontal force profile generated by backstroke swimmers to propel themselves off the wall (de Jesus et al., 2011a; 2013), coaches also recommended that swimmers endeavour to accelerate the centre of mass upwards to clear the water surface because the air presents less resistance than water (de Jesus et al., 2013; Nguyen et al., 2014; Takeda et al., 2014). In fact, the external kinetics involved in backstroke starts should be analysed and interpreted, to consider the magnitude and timing of horizontal and vertical propulsive force vectors applied by the swimmer’s muscular actions to the handgrips and pool wall. Hohmann et al. (2008) and Nguyen et al. (2014) have assessed 3D resultant forces on swimmers’ lower limbs; but only Nguyen et al. (2014) measured the vertical force component. These authors found that altering feet positions at the start resulted in a significant change in peak horizontal and vertical forces. In 2013, FINA approved the use of a new starting platform to prevent the backstroke swimmers sliding down the wall at the start; previously a reasonably common mishap, with disastrous results for the competitor. Therefore, future research analyses are required to ascertain and confirm any advantages that could result from the increased vertical forces backstroke swimmers might achieve and could be translated into a faster racing start.

The instrumented starting blocks used in the previous research referred to the above are few and are now obsolete following the recent FINA facility rule changes approved in 2008 and 2013. The new hand and foot grips now available for backstroke starts have not been instrumented and used in research studies to date. Hence, sport biomechanists and engineers are urged to develop a 3D kinetic system in the new block configuration. Then, one could identify independently how the right and left, upper and lower, limbs contribute to propelling backstroke swimmers during the start.

Beyond the linear kinetics, Green (1987) and Takeda et al. (2014) used angular kinetics principles to study the resistance of the swimmers’ bodies and separated segments to change angular motion during backstroke starts. In previous ventral start studies, swimmers were advised to generate enough angular momentum to make a clean entry into the water (Vantorre et al., 2010). Despite the unique and valid attempt to assess the swimmers’ reluctance to generate angular motion during backstroke start, a number of kinetic and kinematic variables also are required to explain how much rotation is occurring in the sequential starting phases. Takeda et al. (2012) and Takeda et al. (2014) suggested that a combination of kinetic and kinematic measurements are needed for greater clarification of important swimming start components.

### Electromyography (EMG)

As for kinetics, specific EMG studies of swimming starts are few. To measure the muscle activity of backstroke swimmers during the start, a cable EMG system with surface electrodes was used by Hohmann et al. (2008) and de Jesus et al. (2011a; 2011b). This approach requires methodological adaptations to record accurate measurements (Clarys and Cabri, 1993) such as immobilisation of cables and water proofing electrodes. De Jesus et al. (2011a; 2011b) used a complete swimming suit for electrode insulation and cable immobilisation. The full body swimming suit appeared to be suitable for immobilising cables but these had to exit via holes in the suit resulting in potential places for leaks. Further, the use of full body swimming suits is no longer allowed in competition. Insulation to cover electrodes was provided by adhesive bandages (de Jesus et al., 2011a; 2011b; Hohmann et al., 2008). Knowledge of specific muscle activity is an important factor in understanding neuromuscular coordination and effective force production during the different phases of the backstroke start. Overcoming these challenges would greatly assist in determining the most effective techniques and optimise training drills.

The average and integrated EMGs, as amplitude signals, were calculated by Hohmann et al. (2008) and de Jesus et al. (2011a; 2011b), respectively; to provide trunk, and upper and lower limb muscle activation. Muscle intensity data are only one element of motor activity; and the sequential pattern in which the muscles are engaged in a complex backstroke start movement is a more important element (Clarys and Cabri, 1993). In fact, the EMG also provides information on timing of muscle activities in human movements (Bartlett, 2007); nevertheless, only Hohmann et al. (2008) have been concerned about the muscle activation sequence during the backstroke start. According to these authors the backstroke start is initiated by the *Deltoideus Anterior* that had been very active fixing the body in a high set starting position. Despite this initial undertaking, Hohmann’s research group did not provide detailed descriptions of the criteria used to determine the muscles involvement along a continuum from strongly active to an inactive state. The lack of standard methodologies to define the EMG activity makes comparisons between studies difficult.

By studying the sequencing of muscle activation, one can focus on several factors relating to skill; including the timing and overlap of agonist and antagonist activity (Bartlett, 2007). The agonist and antagonist activation in backstroke starts has not been studied yet, due to the swim start acyclic pattern. Nevertheless, Hohmann et al. (2008) mentioned that joint stabilisation occurred during flight and entry phases to overcome the high water resistance. Therefore, simultaneous activation of muscles surrounding joints should be investigated during the backstroke start (Clarys and Cabri, 1993). Seven muscles were commonly studied (Hohmann et al., 2008; de Jesus et al., 2011a, 2011b); namely, the *Biceps Brachii*, *Triceps Brachii*, *Deltoideus Anterior*, *Erector Spinae Longissimus*, *Rectus Femoris*, *Gluteus Maximus* and *Gastrocnemius Medialis*. Authors confirmed the crucial function of the lower limbs to generate the impulse during the take-off phase; however, they disagreed about the main muscle activities of the upper limbs. Studying the above-mentioned biarticular muscles (de Jesus et al., 2011a, 2011b; Hohmann et al., 2008) has highlighted the need to clarify how the mechanical functions vary, depending on the different backstroke start variations and phases (e.g. hip flexor and knee extensor moments for the *Rectus Femoris*). As backstrokers are required to coordinate multiple muscles and joints to propel themselves rigorously out of the pool wall, more studies should couple EMG, kinetic and kinematic approaches to dictate how better backstroke start performance can be achieved.

### Synchronisation methods

The selected studies used a voice command (Stratten, 1970), starting pistol (Rea and Soth, 1967; Miller et al., 1984; Wilson and Howard, 1983), or the official competition timing systems for backstroke start synchronisation (Arellano et al., 2001; Arellano et al., 2003; Chatard et al., 2003; Cornett et al., 2011; Cossor and Mason, 2001; de Jesus et al., 2011a, 2011b, 2013; de Jesus et al., 2012; Girold et al., 2001; Green, 1987; Green et al., 1987; Hohmann et al., 2008; Ikuta et al., 2001; Nguyen et al., 2014; Siljeg et al., 2011; Takeda et al., 2014; Theut and Jensen, 2006).

The competition timing systems were used to simultaneously produce the starting signal and export a light to the video images (Arellano et al., 2001; Arellano et al., 2003; Chatard et al., 2003; Cornett et al., 2011; Cossor and Mason, 2001; de Jesus et al., 2011a; 2013; Hohmann et al., 2008; Ikuta et al., 2001; Nguyen et al., 2014; Siljeg, 2011; Takeda et al., 2014; Theut and Jensen, 2006); and a trigger pulse for the kinetics (de Jesus et al., 2011a; 2013; Hohmann et al., 2008; Nguyen et al., 2014) and EMG synchronisation (de Jesus et al., 2011a; 2011b).

Alternative synchronisation methods have been implemented as the use of force instants to record the swimmer’s handgrip release (de Jesus et al., 2011a; 2013) and feet take-off (de Jesus et al., 2012) for the starting signal definition. Considering that a small temporal and spatial misalignment between different biomechanical devices can lead to large errors in the variables assessed, future studies should use a common system with consistent low trigger delay.

### The backstroke start techniques, variations and main research findings

The main objective of swim-start research has been to identify the most effective start technique in terms of performance (Vantorre et al., 2014). From the selected studies, 65% have established comparisons using backstroke start techniques and variations (Table 1). Researchers have used different distances to assess the effectiveness of each one (Table 3).

Considering the backstroke start studies conducted with variations performed under the NCAA rules, both had used the 6.09 m distance to assess start time. According to Stratten (1970) the most efficient variation was performed when the swimmer’s trunk was positioned upright just in front of the block, and hands holding the horizontal hand-grips; and, the respective mean start time seems to be shorter than the one presented by Rea and Soth (1967). This finding could be explained by the sample sizes and proficiency levels. Rea and Soth (1967) studied one specialist in backstroke start who performed with the trunk inclined forward over the top of the starting block and hands holding a bar mounted over the block. Stratten (1970) included 13 swimmers of different proficiency levels who completed a training period for familiarisation purposes. Yet, it is quite likely that previous experience with a technique may have an impact on start variables and performance (Vantorre et al., 2014). The feet positioned over the pool gutter allowed swimmers to clear the water from the starting position to the beginning of entry by generating greater vertical reaction force; and considered a crucial aspect for better backstroke start performances (de Jesus et al., 2013; Nguyen et al., 2014; Takeda et al., 2014). These statements corroborate other findings where the starts that were performed with shorter horizontal take-off velocities, implied greater aerial trajectory and shorter start time than the variation with a flatter profile (Green et al., 1987) (Table 3).

Most research considered backstroke starts performed under FINA old rules and measured the starting effectiveness using distances from 5 to 15 m (Table 3). Miller et al. (1984) and Arellano et al. (2003) assessed mean start times; although, only the latter specified the set distance. Siljeg et al. (2011) measured the 15 m start time considering the pre and post period of FINA rule changes for feet positioning (FINA 2005–2009, SW. 6.1), which explains the maximum 0.55 s mean difference from the Arellano et al. (2003) findings. Indeed, Nguyen et al. (2014) noted that since the FINA rule changed for feet positioning, many backstrokers have obtained advantages from altering their starting technique to place the feet completely out of the water. To achieve a great start-time performance at 7.5 m, elite backstrokers displayed considerable intraand inter-variability of the upper limbs trajectory during the flight phase (Hohmann et al., 2008; Wilson and Howard, 1983). The upper limb pathways over the centre of mass and close to the body allow the trunk to follow a greater parabolic flight than using a lateral path (Bartlett, 2007; Green, 1987; Maglischo, 2003). According to de Jesus et al. (2013), Nguyen et al. (2014) and Takeda et al. (2014), a greater parabolic flight path assists in minimising drag and optimising propulsion underwater. Since a clear water entry depends on preceding actions performed during the wall and flight phases (Thow et al., 2012), Theut and Jensen (2006) identified the effects of the feet submerged and positioned parallel to each other or staggered (i.e., one above the other) on backstroke start horizontal distance and average velocity. Anecdotal evidence suggested that the feet staggered position prevented swimmers from slipping down the wall; nevertheless, findings did not confirm that difference between variations (Theut and Jensen, 2006). The backstroke start ledge (FINA FR. 2.7, 2013–2017) is pointed out to avoid the slippage; however, further studies are needed to describe in detail how technique must be changed to improve backstroke start performance.

Backstroke starts are performed now under the current FINA rule (adopted in 2005) and only de Jesus et al. (2010; 2011ba; 2011ba; 2013) and Nguyen et al. (2014) compared the variations with the feet parallel, and entirely submerged and out-of-water. Considering the 5 m start time (Table 3) for both variations, shorter values seem to be displayed by the latter research group, which is mainly explained by the swimmers’ greater proficiency level. The variation with feet entirely submerged seems to register lower horizontal take-off mean values in both studies; and the values presented by de Jesus et al. (2013) seem lower than those of Nguyen et al. (2014). Although this finding was not significant, the trend might be explained by the use of a fixed point to indicate the swimmer’s centre of mass. Takeda et al. (2014) verified that backstroke swimmers specialists used a feet-partial-out-of-the-water start, and tended to register greater mean 5 m start time than participants of Nguyen et al. (2014). This might indicate superiority of the variation performed with feet entirely out-of-the-water over the method with partially emerged. De Jesus et al. have not displayed performance differences during above- (2013) and underwater phases (2012), between the variation with feet entirely out and under the water; thereby disagreeing with the Nguyen et al.’s findings (2014). These contradictions might be explained by the larger sample size and greater swimmers’ preference for feet positioned out of the water displayed by Nguyen et al. (2014). De Jesus et al. (2011a; 2013) and Nguyen et al. (2014) stressed that swimmers should generate greater horizontal and vertical take-off velocities when the feet were positioned out of the water to achieve the most appropriate aerial trajectory (de Jesus et al., 2013). The inclusion of the new device for backstroke starts potentially improves the parabolic flight trajectory due to minimised take-off friction force. However, since greater vertical flight trajectory implies deeper water entry, future research should also examine underwater phase variables which can indicate risk of injury, as previously pointed out during youth competitions (Cornett et al., 2011).

## Summary and future directions

The main research findings can be summarised as follows: (1) the phase definitions used in analysing backstroke starts are inconsistent and unclear. Hence, this makes it difficult to determine how many changes over time can be attributed to a real change, or mere differences between definitions; (2) studies conducted in laboratory settings have adopted kinematics, kinetics and EMG; however, many research challenges remain in both settings to improve the methods of quantifying valid, reliable and accurate data; (3) the temporal variables, particularly the starting time, were most studied; and backstroke start movements were predominantly described using linear kinematics; (4) most of the experimental and competition research findings are now out of date since the backstroke start rules have been recently changed, and the studies were completed under swimming rules which are now obsolete.

Future research would help coaches and swimmers by exploring issues not yet fully addressed in the literature. For example: (1) determination of a consistent observational model for categorisation and study of the backstroke start technique; (2) development of appropriate biomechanical measurements and research methodologies as standard tools; for scientific purposes and training support, competition preparation and analysis; (3) reinforcement of more holistic and process-oriented biomechanical approaches in laboratory procedures: involving interactions of kinematics, kinetics and EMG variables; from aerial, aerial/in-water and underwater phases; definitions for more detailed parameters which better describe the overall backstroke start in competitions, beyond the starting time; (4) focusing on studies based on the actual FINA rules and the new starting block configurations.

## Figures and Tables

**Table 1:** Descriptive analysis of the 22 included studies with the authors, main aim, swimmer’s sample proficiency and data collection setting

Author (s)	Main aim	Proficiency	Setting
Rea and Soth (1967)	Comparison of two NCAA variations	Olympic	Experimental
Stratten (1970)	Comparison of FINA and NCAA techniques	Recreational to Olympic	Experimental
Wilson and Howard (1983)	FINA backstroke start clusters	State to Olympic	Experimental
Miller et al. (1984)	Comparison of FINA technique	International	Competition
Green (1987)	Comparison of NCAA variations	National	Experimental
Green et al. (1987)	Comparison of NCAA variations	State	Experimental
Arellano et al. (2001)	Determinant swimming event factors	Olympic	Competition
Cossor and Mason (2001)	Correlation of FINA phases and starting time	Olympic	Competition
Girold et al. (2001)	Comparison among 200 m proficiency levels	Olympic	Competition
Ikuta et al. (2001)	Comparison between Japanese and other nations	Olympic	Competition
Arellano et al. (2003)	Correlation of FINA start and 100 m event time	International	Competition
Chatard et al. (2003)	Comparison among 200 m proficiency levels	Olympic	Competition
Theut and Jensen (2006)	Comparison of two FINA variations	Not clearly defined	Experimental
Hohmann et al. (2008)	FINA inter and intra-individual variability	International	Experimental
de Jesus et al. (2010)	Comparison of two FINA variations	National	Experimental
de Jesus et al. (2011a)	Performance prediction for two FINA variations	National	Experimental
de Jesus et al.(2011b)	Comparison of two FINA starting phases	National	Experimental
Siljeg et al. (2011)	Comparison of 100 m starting performance	International	Competition
Cornett et al. (2011)	Racing start safety analysis	Not clearly defined	Competition
de Jesus et al. (2012)	Comparison of two FINA variations	National	Experimental
de Jesus et al. (2013)	Comparison of two FINA variations	National	Experimental
Takeda et al. (2014)	Comparison between specialists and non-specialists	National	Experimental
Nguyen et al. (2014)	Comparison of two FINA variations	National	Experimental

**Table 2:** The kinematic parameters studied at the overall starting and during the hands-off, take-off and flight phases.

Authors	Overall	Hands-off	Take-off	Flight
Rea and Soth (1967)	Temporal, velocity	/	/	/
Stratten (1970)	Temporal	/	Temporal	/
Wilson and Howard (1983)	/	Segmental length, angle	Segmental length, angle	Segmental length, angle
Miller et.al. (1984)	Temporal and distance	Temporal	Temporal, distance	Temporal
Green (1987)	Centre of mass displacement	Joint angles, centre of mass velocity, acceleration, angular velocity	Joint angles, centre of mass velocity, acceleration, angular velocity	Joint angles, centre of mass velocity, acceleration, angular velocity
Green et al. (1987)	Temporal	/	/	/
Arellano et al. (2001)	Temporal	/	/	/
Cossor and Mason (2001)	Temporal	/	Temporal	Temporal, distance
Girold et al.(2001)	Temporal, velocity	/	/	/
Ikuta et al.(2001)	Temporal	/	/	/
Arellano et al.(2003)	Temporal, velocity	/	/	/
Chatard et al. (2003)	Velocity	/	/	/
Theut and Jensen (2006)	Velocity, distance	/	/	/
Hohmann et al.(2008)	Temporal	Temporal	Temporal, velocity	Temporal
de Jesus et al.(2010)	Temporal Angular displacement and velocity	Temporal, centre of mass displacement and velocity	Temporal, centre of mass displacement	Temporal, centre of mass displacement,
de Jesus et al. (2011a)	Temporal	Centre of mass positioning and velocity	Centre of mass displacement, velocity, angle	Centre of mass velocity
de Jesus et al. (2011b)	/	/	/	/
de Jesus et al. (2012)	/	/	/	/
Cornett et al. (2011)	/	/	/	/
Siljeg et al.(2011)	Temporal	/	/	/
de Jesus et al. (2013)	Temporal	Centre of mass position and velocity	Centre of mass velocity, angle	Centre of mass velocity, angle
Takeda et al., (2014)	Temporal	Height of toe, angular velocity	Temporal, Centre of mass velocity, joint angles, angular velocity	/
Nguyen et al. (2014)	Temporal	/	Temporal, displacement, velocity	/

**Table 3:** The set distance for the backstroke start variations performance assessment

Authors	Backstroke start variations (feet positioning)	Distance (m)	Start time (s)	Take-off Velocity (m.s^−1^)
Rea and Soth (1967)	Entirely emerged, toes over the gutter	6.09	2.69	-
Rea and Soth (1967)	Entirely emerged, toes over the gutter, trunk leaned on block	6.09	2.51	-
Stratten (1970)	Entirely immersed	6.09	2.48	-
Stratten (1970)	Entirely emerged, toes curled over the pool gutter	6.09	2.26	-
Stratten (1970)	Entirely emerged, toes over the gutter, trunk leaned on block	6.09	2.49	-
Miller et al. (1984)	Entirely immersed	-	3.58	
Green et al. (1987)	Entirely emerged, toes over the gutter	22.86	16.62	4.70
Green et al. (1987)	Entirely emerged, toes over the gutter, parabolic flight trajectory	22.86	17.0	3.62
Arellano et al. (2003)	Entirely immersed	15	8.27	-
Hohmann et al. (2008)	Entirely immersed	7.5	3.29	3.45
de Jesus et al. (2010)	Entirely immersed	-	0.93	-
de Jesus et al. (2010)	Entirely emerged	-	0.98	-
Siljeg et al. (2011)	Entirely immersed	15	8.30	-
Siljeg et al. (2011)	-	15	7.72	-
de Jesus et al. (2013)	Entirely immersed	5	1.96	3.29
de Jesus et al. (2013)	Entirely emerged	5	2.11	3.80
Takeda et al. (2014)	Partially immersed	5	1.89	3.76
Nguyen et al. (2014)	Entirely immersed	5/ 15	1.86 / 7.59	3.51
Nguyen et al. (2014)	Entirely emerged	5/ 15	1.72 / 7.51	3.65
